# A Pilot Program of Virtual Healthcare Delivery to Older Veterans

**DOI:** 10.1093/geroni/igaf122.3147

**Published:** 2025-12-31

**Authors:** Sangeeta Rana, Michelle Rossi, Stephanie Sodders, Tammy Knell, Coleen Cardamone

**Affiliations:** VA Pittsburgh Health Care System, Pittsburgh, Pennsylvania, United States; VA Pittsburgh Health Care System, Pittsburgh, Pennsylvania, United States; VA Pittsburgh Health Care System, Pittsburgh, Pennsylvania, United States; VA Pittsburgh Health Care System, Pittsburgh, Pennsylvania, United States; VA Pittsburgh Health Care System, Pittsburgh, Pennsylvania, United States

## Abstract

Telemedicine and ECHO® programs have successfully assisted in delivering specialized medical care as part of outreach efforts to rural and underserved areas. We describe a new pilot, Tele Geriatric Evaluation & Management program (TeleGEM) undertaken by our GRECC (Geriatric Research Education and Clinical Center) using telemedicine to deliver an interdisciplinary team-based model of Comprehensive Geriatric Assessment to Veterans in the network who lacked access to this service. Our team included a geriatrician, registered nurse, pharmacist, and administrative assistant with rehab services and neuropsychology available on a consultative basis. Thirty-three frail Veterans,65y & older (average 77y) were referred to our program for evaluation during a 10-month period starting January 2024. Telemedicine technologies used to provide this service from the tertiary care facility included the use of telemedicine carts available at the local VA medical centers (67%), in Veterans’ homes using audiovisual digital devices and/or audio visits by telephone (24%), in addition to E-consults (9%) completed by chart reviews. Veterans were assessed using the 5M framework: what Matters, Mind, Multicomplexity, Medications, and Mobility. Other measures included mileage saved by avoiding travel to the geriatric clinics located at the tertiary care facility. The Tele GEM platform can serve as a novel and valuable healthcare delivery model of care providing comprehensive geriatric assessments which can help improve function and quality of life for frail older Veterans, thus reducing the burden of travel to tertiary care centers.

